# Supplementary material for: Translation initiation or elongation inhibition triggers contrasting effects on Caenorhabditis elegans survival during pathogen infection
Source: mBio. 2024 Sep 30;15(11):e02485-24. doi: 10.1128/mbio.02485-24 (PMC11559039; doi:10.1128/mbio.02485-24)
Supplement: Supplemental material — Fig. S1 to S10, Table S1, and Table S10. [file mbio.02485-24-s0001.pdf]

Supplementary Information for

Translation initiation or elongation inhibition triggers contrasting effects on *Caenorhabditis elegans* survival during pathogen infection

Annesha Ghosh[1] and Jogender Singh[1,*]

[1]Department of Biological Sciences, Indian Institute of Science Education and Research, Mohali, Punjab, 140306, India

[*]For correspondence: jogender@iisermohali.ac.in

Supplementary Figures

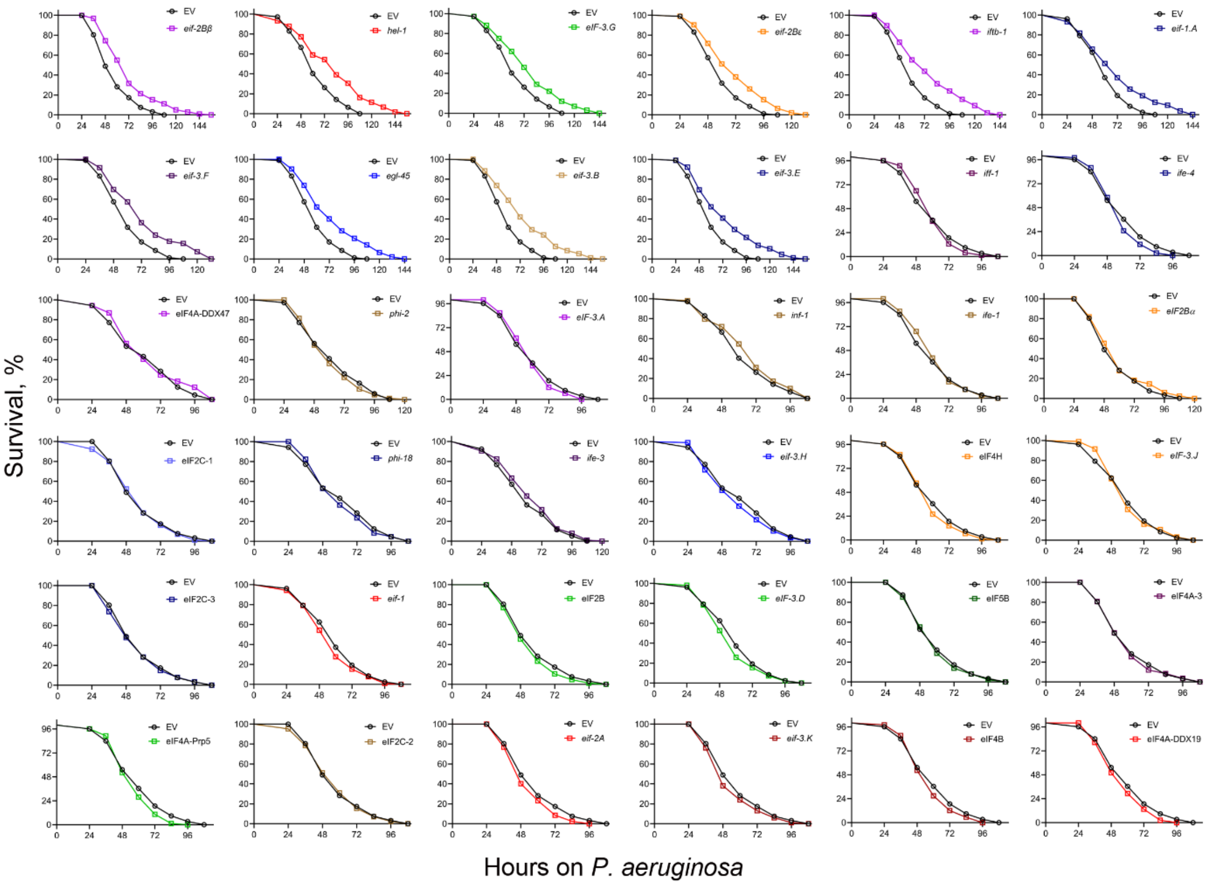

Figure S1. Inhibition of several translation initiation factors improves *C. elegans* survival on *P. aeruginosa*

Representative survival plots of N2 animals on *P. aeruginosa* PA14 at 25°C after treatment with the empty vector (EV) control and RNAi against various translation initiation factors. The EV control is common for different cohorts of the survival curves. Detailed statistical analysis is available in Table S2.

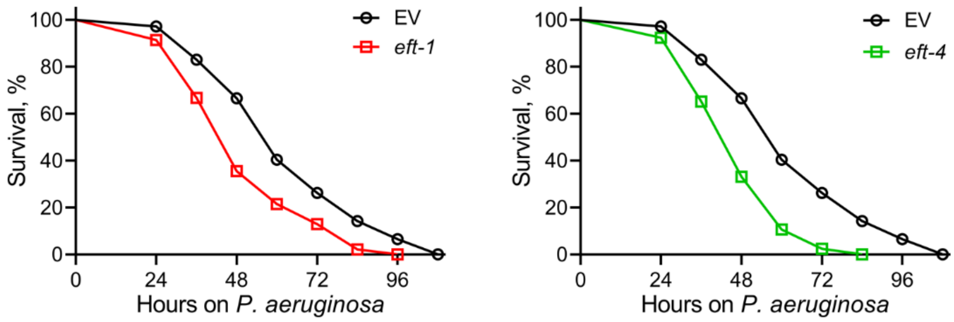

Figure S2. Inhibition of translation elongation factors reduces *C. elegans* survival on *P. aeruginosa*

Representative survival plots of N2 animals on *P. aeruginosa* PA14 at 25°C after treatment with the empty vector (EV) control, *eft-1*, and *eft-4* RNAi. The EV control is common for the two survival plots. Detailed statistical analysis is available in Table S2.

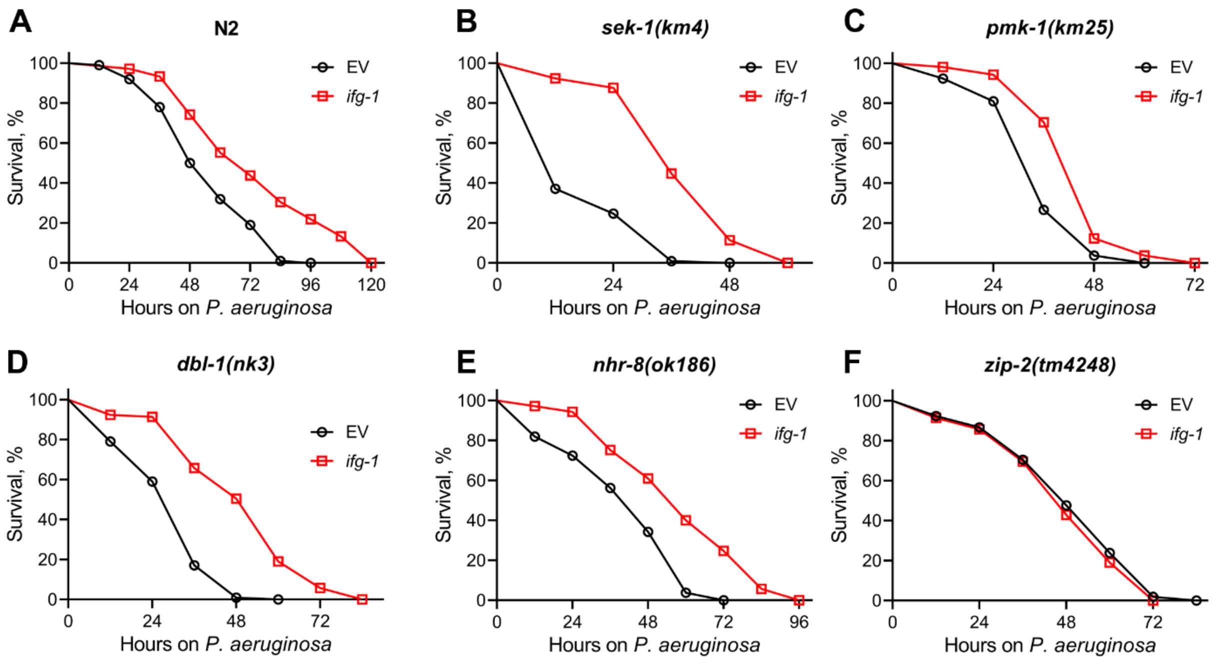

Figure S3. Knockdown of *ifg-1* increases *C. elegans* survival on *P. aeruginosa* via transcription factor ZIP-2

Representative survival plots of N2 (A), *sek-1(km4)* (B), *pmk-1(km25)* (C), *dbl-1(nk3)* (D), *nhr-8(ok186)* (E), and *zip-2(tm4248)* (F) animals on *P. aeruginosa* PA14 at 25°C after treatment with the empty vector (EV) control and *ifg-1* RNAi. *P*<0.001 for *ifg-1* RNAi compared to EV control for N2, *sek-1(km4)*, *pmk-1(km25)*, *dbl-1(nk3)*, and *nhr-8(ok186)* animals. Survival curves for *ifg-1* RNAi compared to the EV control for *zip-2(tm4248)* animals are nonsignificant.

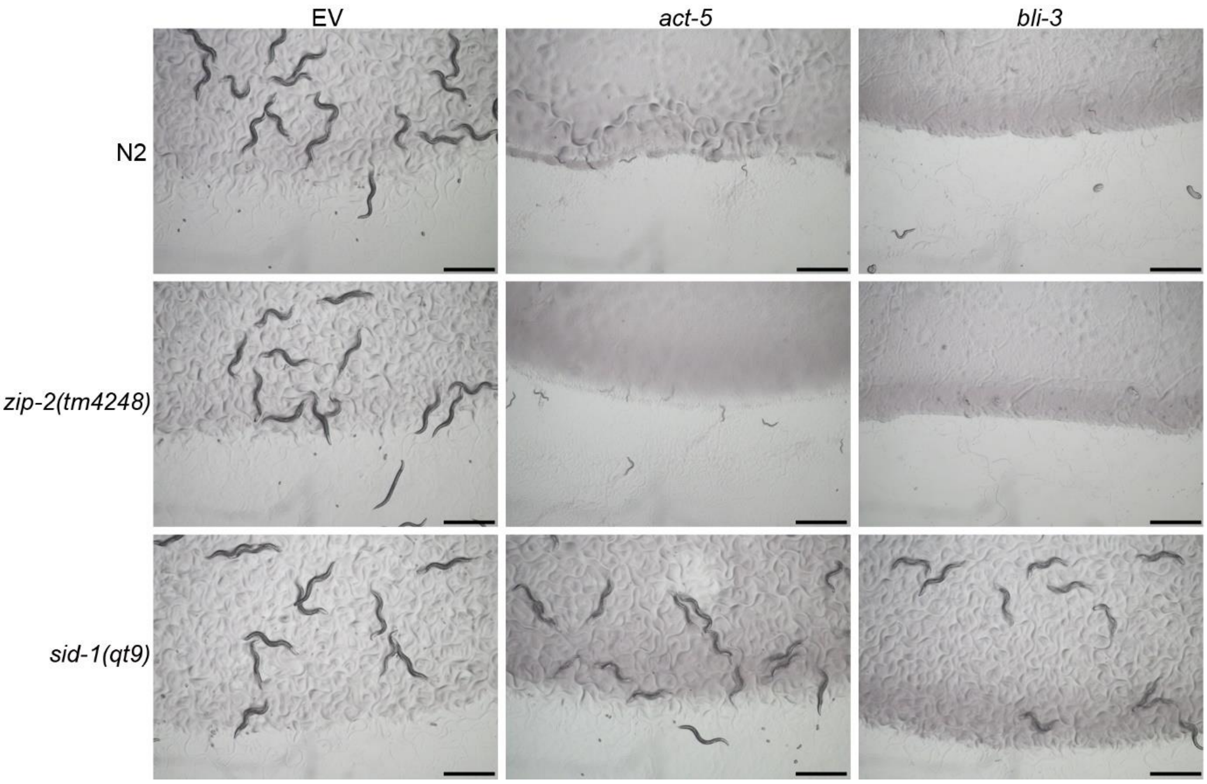

Figure S4. The *zip-2(tm4248)* animals are sensitive to RNAi

Representative images of wild-type N2, *zip-2(tm4248)*, and *sid-1(qt9)* worms on the empty vector (EV) control, *act-5*, and *bli-3* RNAi. Scale bar = 1 mm.

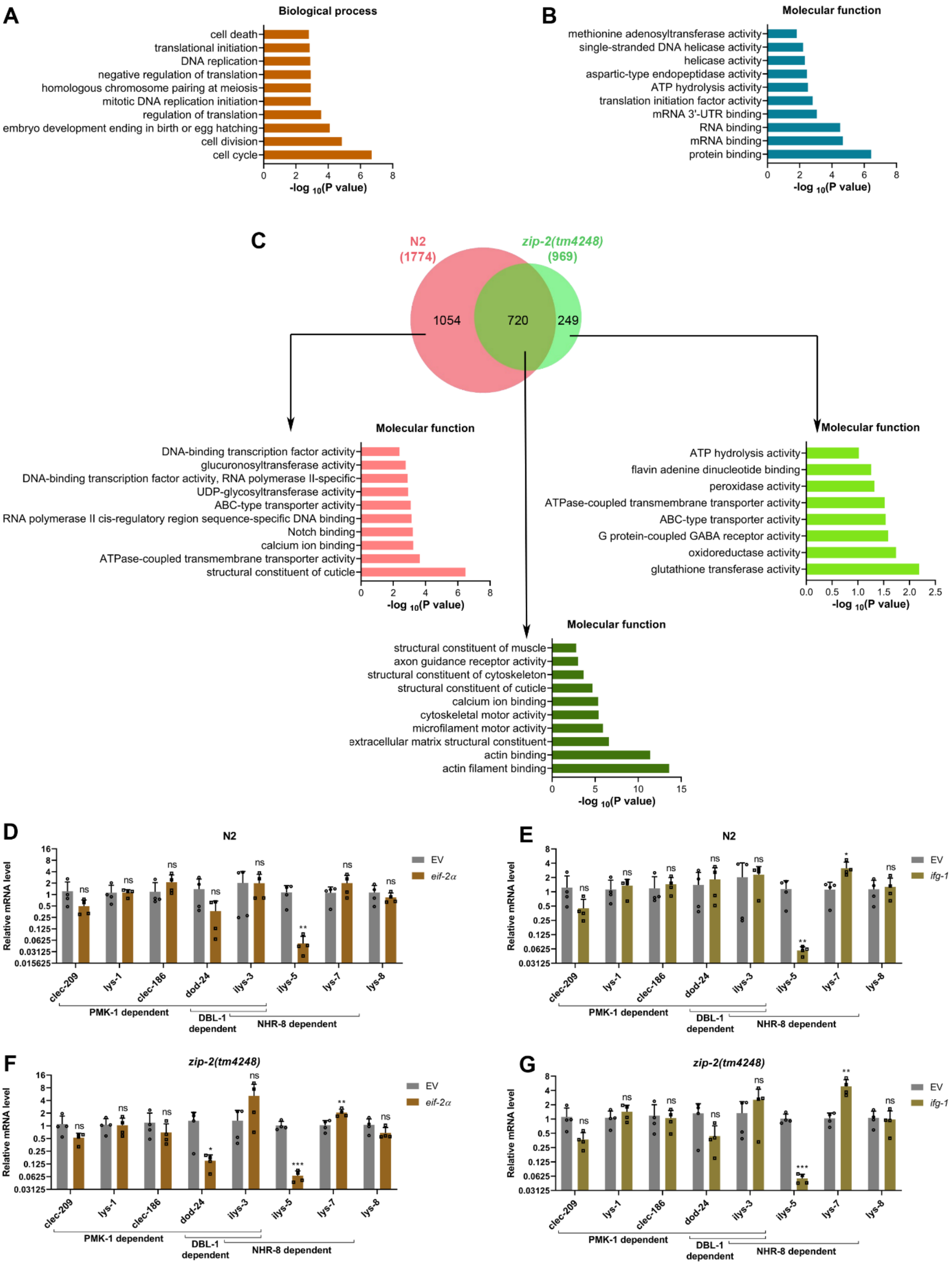

Figure S5. Inhibition of translation initiation activates a ZIP-2-dependent immune response

(A)-(B) Gene Ontology (GO) enrichment analysis of downregulated genes upon *eif-2α* knockdown in N2 animals for biological processes (A) and molecular functions (B).

(C) Venn diagram showing the overlap between genes upregulated upon *eif-2α* knockdown in N2 and *zip-2(tm4248)* animals. The GO analysis for molecular functions of unique and common genes is shown.
(D) Quantitative reverse transcription-PCR (qRT-PCR) for immune genes expression analysis of N2 animals after treatment with the empty vector (EV) control and *eif-2α* RNAi.
(E) qRT-PCR for immune genes expression analysis of N2 animals after treatment with the EV control and *ifg-1* RNAi.
(F) qRT-PCR for immune genes expression analysis of *zip-2(tm4248)* animals after treatment with the EV control and *eif-2α* RNAi.
(G) qRT-PCR for immune genes expression analysis of *zip-2(tm4248)* animals after treatment with the EV control and *ifg-1* RNAi.
For panels (D)-(G), ***$P < 0.001$, **$P < 0.01$, and *$P < 0.05$ via the *t*-test. ns, nonsignificant. Data represent the mean and standard deviation from four independent experiments.

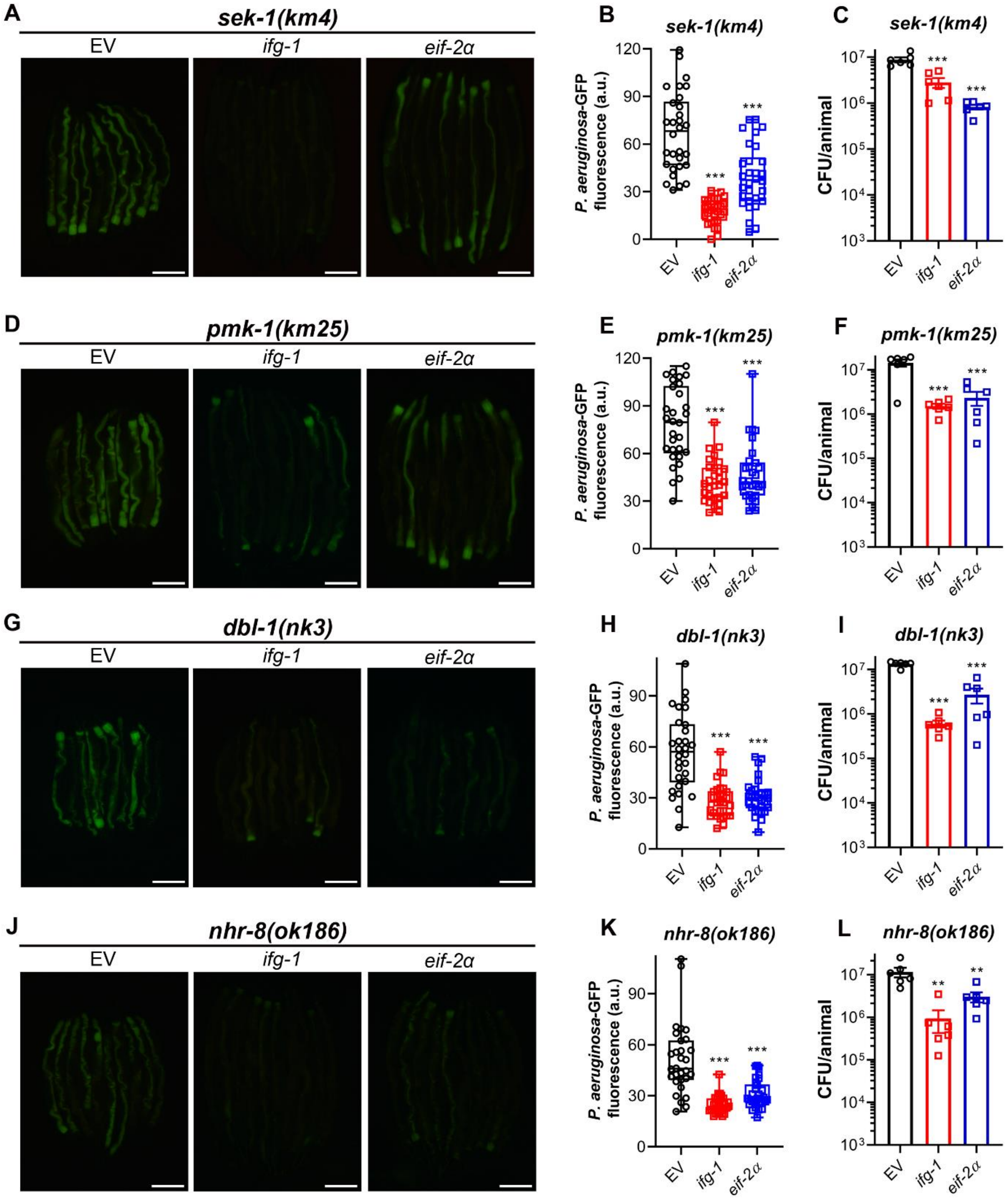

Figure S6. Inhibition of translation initiation reduces *P. aeruginosa* colonization in *sek-1(km4)*, *pmk-1(km25)*, *dbl-1(nk3)*, and *nhr-8(ok186)* animals

(A) Representative fluorescence images of *sek-1(km4)* animals incubated on *P. aeruginosa*-GFP for 12 hours at 25°C after treatment with the empty vector (EV) control, *ifg-1*, and *eif-2α* RNAi. Scale bar = 200 µm.

(B) Quantification of GFP levels of *sek-1(km4)* animals incubated on *P. aeruginosa*-GFP for 12 hours at 25°C after treatment with the EV control, *ifg-1*, and *eif-2α* RNAi. ***$P < 0.001$

via ordinary one-way ANOVA followed by Dunnett's multiple comparisons test ($n = 30$ worms each).

(C) Colony-forming units (CFU) per animal of *sek-1(km4)* animals incubated on *P. aeruginosa*-GFP for 12 hours at 25°C after treatment with the EV control, *ifg-1*, and *eif-2α* RNAi. ***$P < 0.001$ via ordinary one-way ANOVA followed by Dunnett's multiple comparisons test ($n = 6$ biological replicates).

(D) Representative fluorescence images of *pmk-1(km25)* animals incubated on *P. aeruginosa*-GFP for 24 hours at 25°C after treatment with the EV control, *ifg-1*, and *eif-2α* RNAi. Scale bar = 200 µm.

(E) Quantification of GFP levels of *pmk-1(km25)* animals incubated on *P. aeruginosa*-GFP for 24 hours at 25°C after treatment with the EV control, *ifg-1*, and *eif-2α* RNAi. ***$P < 0.001$ via ordinary one-way ANOVA followed by Dunnett's multiple comparisons test ($n = 30$ worms for EV control and *eif-2α* RNAi, and 29 worms for *ifg-1* RNAi).

(F) CFU per animal of *pmk-1(km25)* animals incubated on *P. aeruginosa*-GFP for 24 hours at 25°C after treatment with the EV control, *ifg-1*, and *eif-2α* RNAi. ***$P < 0.001$ via ordinary one-way ANOVA followed by Dunnett's multiple comparisons test ($n = 6$ biological replicates).

(G) Representative fluorescence images of *dbl-1(nk3)* animals incubated on *P. aeruginosa*-GFP for 24 hours at 25°C after treatment with the EV control, *ifg-1*, and *eif-2α* RNAi. Scale bar = 200 µm.

(H) Quantification of GFP levels of *dbl-1(nk3)* animals incubated on *P. aeruginosa*-GFP for 24 hours at 25°C after treatment with the EV control, *ifg-1*, and *eif-2α* RNAi. ***$P < 0.001$ via ordinary one-way ANOVA followed by Dunnett's multiple comparisons test ($n = 30$ worms each).

(I) CFU per animal of *dbl-1(nk3)* animals incubated on *P. aeruginosa*-GFP for 24 hours at 25°C after treatment with the EV control, *ifg-1*, and *eif-2α* RNAi. ***$P < 0.001$ via ordinary one-way ANOVA followed by Dunnett's multiple comparisons test ($n = 6$ biological replicates).

(J) Representative fluorescence images of *nhr-8(ok186)* animals incubated on *P. aeruginosa*-GFP for 24 hours at 25°C after treatment with the EV control, *ifg-1*, and *eif-2α* RNAi. Scale bar = 200 µm.

(K) Quantification of GFP levels of *nhr-8(ok186)* animals incubated on *P. aeruginosa*-GFP for 24 hours at 25°C after treatment with the EV control, *ifg-1*, and *eif-2α* RNAi. ***$P < 0.001$ via ordinary one-way ANOVA followed by Dunnett's multiple comparisons

test ($n = 30$ worms each).

(L) CFU per animal of *nhr-8(ok186)* animals incubated on *P. aeruginosa*-GFP for 24 hours at 25°C after treatment with the EV control, *ifg-1*, and *eif-2α* RNAi. **$P < 0.01$ via ordinary one-way ANOVA followed by Dunnett's multiple comparisons test ($n = 6$ biological replicates).

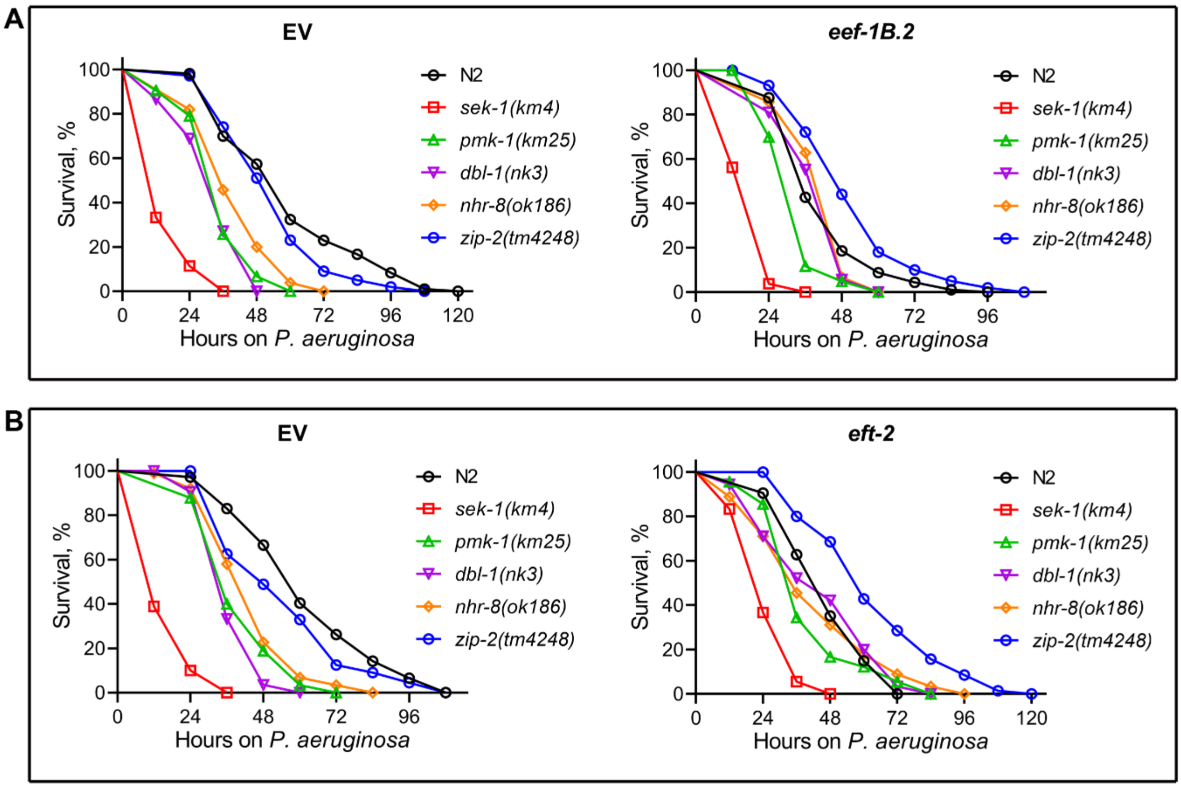

Figure S7. Detrimental effects of knockdown of *eef-1B.2* and *eft-2* on *C. elegans* survival on *P. aeruginosa* diminish in *zip-2(tm4248)* animals

(A) Representative survival plots of worm strains on *P. aeruginosa* PA14 at 25°C after treatment with the empty vector (EV) control RNAi (left panel) and *eef-1B.2* RNAi (right panel).

(B) Representative survival plots of worm strains on *P. aeruginosa* PA14 at 25°C after treatment with the EV control RNAi (left panel) and *eft-2* RNAi (right panel).

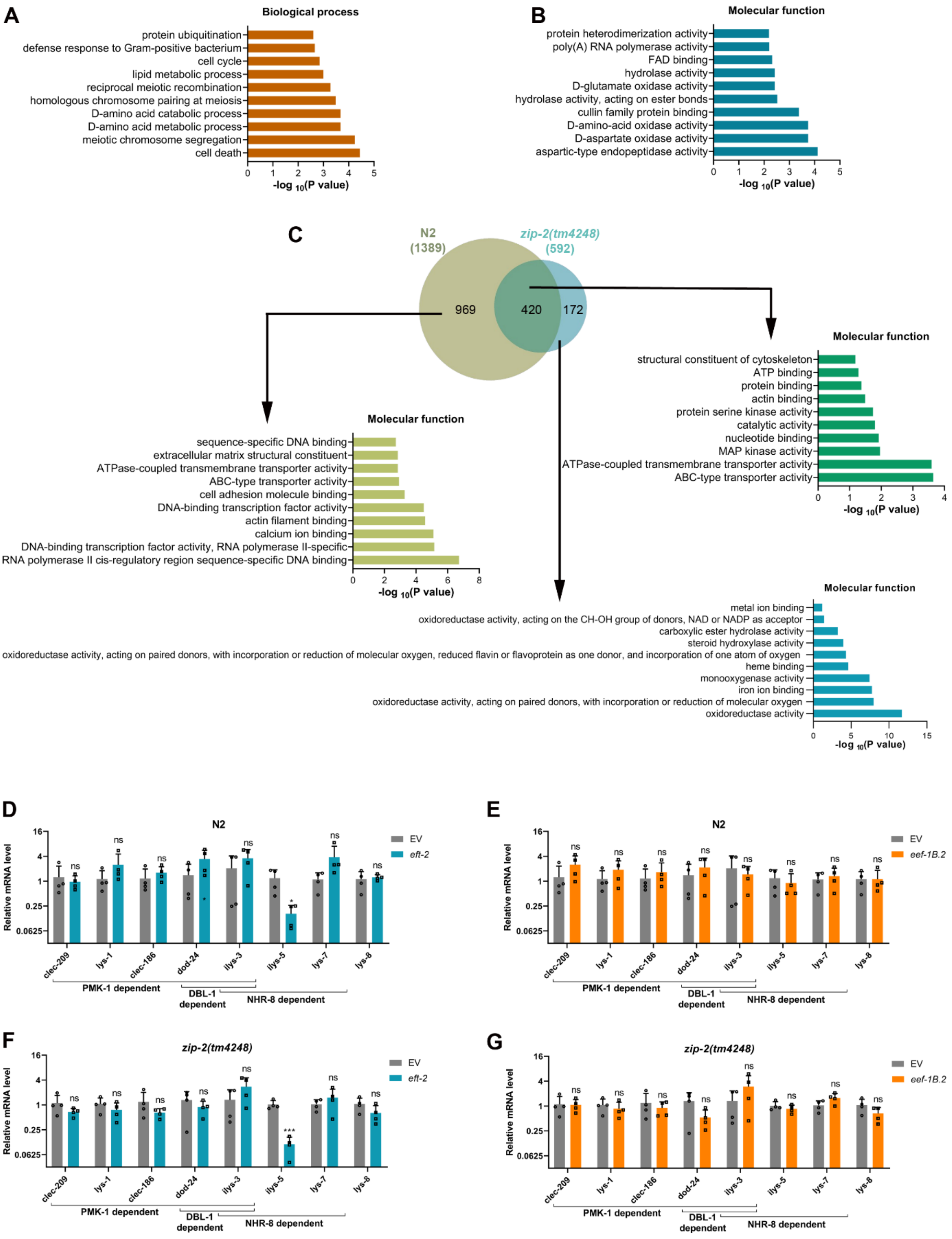

Figure S8. Inhibition of translation elongation activates ZIP-2-dependent and ZIP-2-independent immune responses

(A)-(B) Gene Ontology (GO) enrichment analysis of downregulated genes upon *eft-2* knockdown in N2 animals for biological processes (A) and molecular functions (B).

(C) Venn diagram showing the overlap between genes upregulated upon *eft-2* knockdown in

N2 and *zip-2(tm4248)* animals. The GO analysis for molecular functions of unique and common genes is shown.

(D) Quantitative reverse transcription-PCR (qRT-PCR) for immune genes expression analysis of N2 animals after treatment with the empty vector (EV) control and *eft-2* RNAi.

(E) qRT-PCR for immune genes expression analysis of N2 animals after treatment with the EV control and *eef-1B.2* RNAi.

(F) qRT-PCR for immune genes expression analysis of *zip-2(tm4248)* animals after treatment with the EV control and *eft-2* RNAi.

(G) qRT-PCR for immune genes expression analysis of *zip-2(tm4248)* animals after treatment with the EV control and *eef-1B.2* RNAi.

For panels (D)-(G), ***$P < 0.001$ and *$P < 0.05$ via the *t*-test. ns, nonsignificant. Data represent the mean and standard deviation from four independent experiments.

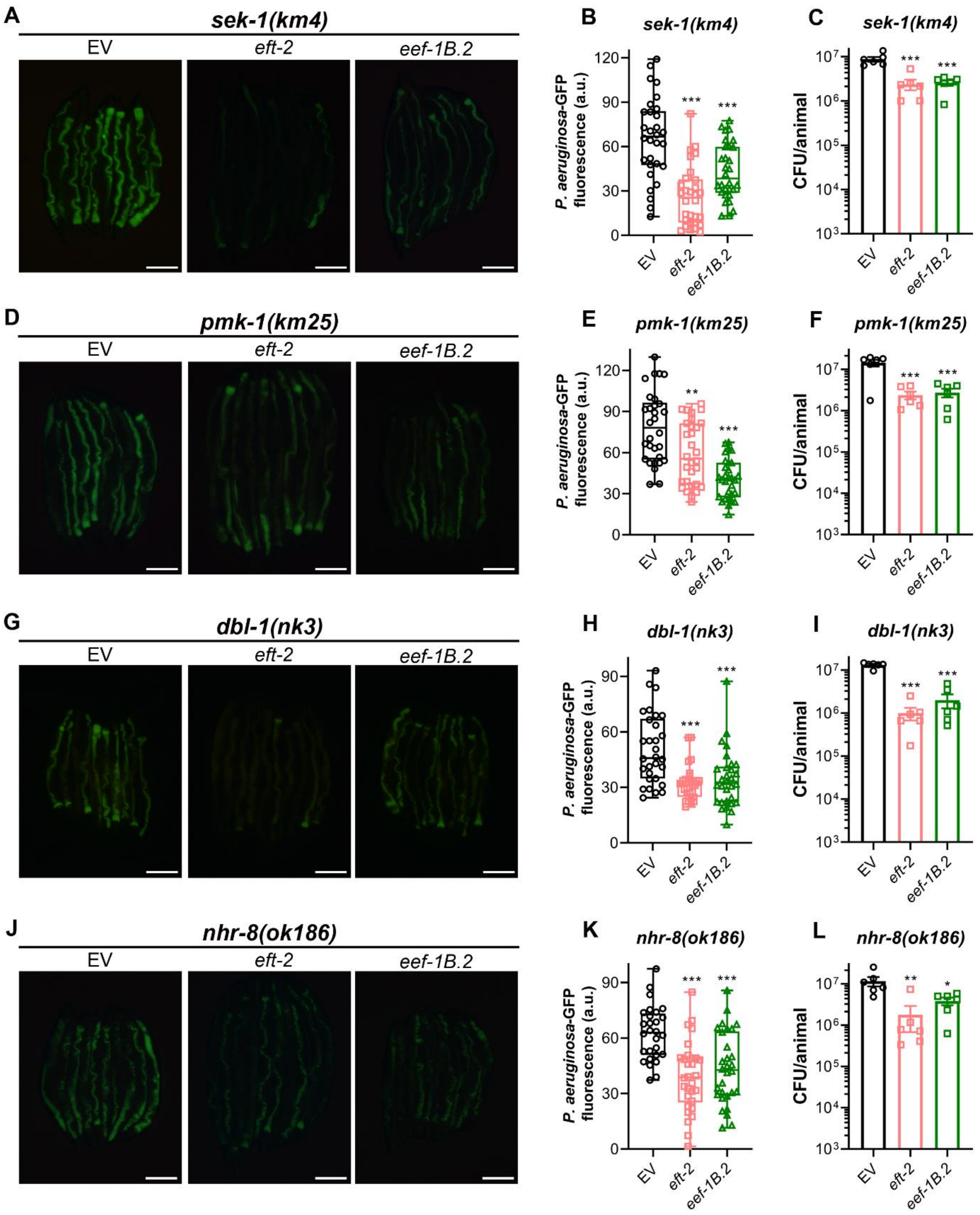

Figure S9. Inhibition of translation elongation reduces *P. aeruginosa* colonization in *sek-1(km4)*, *pmk-1(km25)*, *dbl-1(nk3)*, and *nhr-8(ok186)* animals

(A) Representative fluorescence images of *sek-1(km4)* animals incubated on *P. aeruginosa*-GFP for 12 hours at 25°C after treatment with the empty vector (EV) control, *eft-2*, and *eef-1B.2* RNAi. Scale bar = 200 µm.

(B) Quantification of GFP levels of *sek-1(km4)* animals incubated on *P. aeruginosa*-GFP for 12 hours at 25°C after treatment with the EV control, *eft-2*, and *eef-1B.2* RNAi. ***$P < 0.001$ via ordinary one-way ANOVA followed by Dunnett's multiple comparisons test ($n = 30$ worms each).

(C) Colony-forming units (CFU) per animal of *sek-1(km4)* animals incubated on *P. aeruginosa*-GFP for 12 hours at 25°C after treatment with the EV control, *eft-2*, and *eef-1B.2* RNAi. ***$P < 0.001$ via ordinary one-way ANOVA followed by Dunnett's multiple comparisons test ($n = 6$ biological replicates).

(D) Representative fluorescence images of *pmk-1(km25)* animals incubated on *P. aeruginosa*-GFP for 24 hours at 25°C after treatment with the EV control, *eft-2*, and *eef-1B.2* RNAi. Scale bar = 200 μm.

(E) Quantification of GFP levels of *pmk-1(km25)* animals incubated on *P. aeruginosa*-GFP for 24 hours at 25°C after treatment with the EV control, *eft-2*, and *eef-1B.2* RNAi. ***$P < 0.001$ and **$P < 0.01$ via ordinary one-way ANOVA followed by Dunnett's multiple comparisons test ($n = 30$ worms each).

(F) CFU per animal of *pmk-1(km25)* animals incubated on *P. aeruginosa*-GFP for 24 hours at 25°C after treatment with the EV control, *eft-2*, and *eef-1B.2* RNAi. ***$P < 0.001$ via ordinary one-way ANOVA followed by Dunnett's multiple comparisons test ($n = 6$ biological replicates).

(G) Representative fluorescence images of *dbl-1(nk3)* animals incubated on *P. aeruginosa*-GFP for 24 hours at 25°C after treatment with the EV control, *eft-2*, and *eef-1B.2* RNAi. Scale bar = 200 μm.

(H) Quantification of GFP levels of *dbl-1(nk3)* animals incubated on *P. aeruginosa*-GFP for 24 hours at 25°C after treatment with the EV control, *eft-2*, and *eef-1B.2* RNAi. ***$P < 0.001$ via ordinary one-way ANOVA followed by Dunnett's multiple comparisons test ($n = 30$ worms each).

(I) CFU per animal of *dbl-1(nk3)* animals incubated on *P. aeruginosa*-GFP for 24 hours at 25°C after treatment with the EV control, *eft-2*, and *eef-1B.2* RNAi. ***$P < 0.001$ via ordinary one-way ANOVA followed by Dunnett's multiple comparisons test ($n = 6$ biological replicates).

(J) Representative fluorescence images of *nhr-8(ok186)* animals incubated on *P. aeruginosa*-GFP for 24 hours at 25°C after treatment with the EV control, *eft-2*, and *eef-1B.2* RNAi. Scale bar = 200 μm.

(K) Quantification of GFP levels of *nhr-8(ok186)* animals incubated on *P. aeruginosa*-GFP

for 24 hours at 25°C after treatment with the EV control, *eft-2*, and *eef-1B.2* RNAi. ***$P < 0.001$ via ordinary one-way ANOVA followed by Dunnett's multiple comparisons test ($n = 30$ worms for *eft-2* and *eef-1B.2* RNAi, and 28 worms for EV control).

(L) CFU per animal of *nhr-8(ok186)* animals incubated on *P. aeruginosa*-GFP for 24 hours at 25°C after treatment with the EV control, *eft-2*, and *eef-1B.2* RNAi. **$P < 0.01$ and *$P < 0.05$ via ordinary one-way ANOVA followed by Dunnett's multiple comparisons test ($n = 6$ biological replicates).

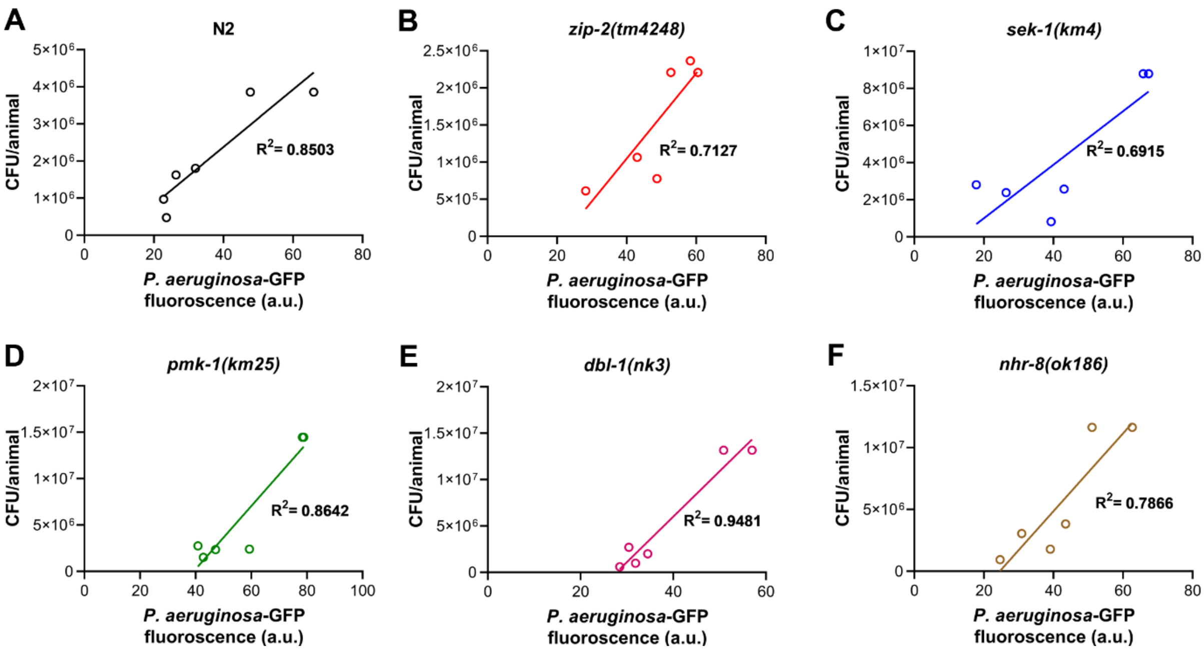

Figure S10. *C. elegans* gut *P. aeruginosa*-GFP levels correlate with colony-forming units (CFU) per animal

Correlation between *P. aeruginosa*-GFP levels per animal and CFU per animal for N2 (A), *zip-2(tm4248)* (B), *sek-1(km4)* (C), *pmk-1(km25)* (D), *dbl-1(nk3)* (E), and *nhr-8(ok186)* (F).

Table S1: The list of *C. elegans* translation factors available in the Ahringer RNAi library and the effects of their knockdown on the development and fertility of N2 wild-type worms

**Initiation factors**		
**Predicted translation factors**	**Gene ID**	**Development/fertility**
eif-1	T27F7.3	L4
eif-1.A	H06H21.3	Late L4/young adults
eif-2A	E04D5.1	Adults
eif-2α	Y37E3.10	L3-L4
iftb-1	K04G2.1	L3
eIF2B	C01G10.9	Adults
eif-2Bα	ZK1098.4	Adults
eif-2Bβ	Y47H9C.7	Adults
eif-2Bε	D2085.3	L4
eif-2Bγ	C15F1.4	Adults (sterile)
eIF2C-1	T22B3.2	Adults
eIF2C-2	ZK757.3	Adults
eIF2C-3	R09A1.1	Adults
egl-45	C27D11.1	L4
eif-3.A	F55H2.6	Adults
eif-3.B	Y54E2A.11	L3
eif-3.C	T23D8.4	Late L4/young adults
eif-3.D	R08D7.3	L3
eif-3.E	B0511.10	L4
eif-3.F	D2013.7	L3
eif-3.G	F22B5.2	L3
eif-3.H	C41D11.2	Late L4/Adults
eif-3.J	Y40B1B.5	L4
eif-3.K	T16G1.11	Adults
inf-1	F57B9.6	L3
phi-2	F57B9.3	L4
eIF4A-3	F33D11.10	Adults
eIF4A-p56	C07H6.5	Adults (sterile)
hel-1	C26D10.2	L4
eIF4A-DDX47	T26G10.1	L4
eIF4A-DDX19	T07D4.4	Adults
eIF4A-Prp5	F53H1.1	Adults
eIF4B	Y73B6BL.33	Adults
ife-1	F53A2.6	Adults
ife-2	R04A9.4	Adults
ife-3	B0348.6	Adults
ife-4	C05D9.5	Adults
ifg-1	M110.4	L3
eIF4H	T12D8.2	Adults

phi-18	C37C3.2	L4
iff-1	T05G5.10	Adults
iff-2	F54C9.1	Adults (sterile)
eIF5B	Y54F10BM.2	Adults
**Elongation factors**		
**Predicted translation factors**	**Gene ID**	**Development/fertility**
eft-4	R03G5.1	L3-L4
eef-1B.2	Y41E3.10	Adults
eft-2	F25H5.4	L3
eft-1	ZK328.2	L3

Table S2: Statistical analysis of survival curves from three independent experiments for each condition. A separate Excel file is provided.

Table S3: Upregulated and downregulated genes in *eif-2α* RNAi versus empty vector control RNAi in N2 animals. Genes exhibiting at least two-fold change and *P*-value <0.01 were considered differentially expressed. A separate Excel file is provided.

Table S4: Upregulated and downregulated genes in *eif-2α* RNAi versus empty vector control RNAi in *zip-2(tm4248)* animals. Genes exhibiting at least two-fold change and *P*-value <0.01 were considered differentially expressed. A separate Excel file is provided.

Table S5: Comparision of genes upregulated upon *eif-2α* RNAi in N2 and *zip-2(tm4248)* animals. A separate Excel file is provided.

Table S6: Upregulated and downregulated genes in *eft-2* RNAi versus empty vector control RNAi in N2 animals. Genes exhibiting at least two-fold change and *P*-value <0.01 were considered differentially expressed. A separate Excel file is provided.

Table S7: Upregulated and downregulated genes in *eft-2* RNAi versus empty vector control RNAi in *zip-2(tm4248)* animals. Genes exhibiting at least two-fold change and *P*-value <0.01 were considered differentially expressed. A separate Excel file is provided.

Table S8: Comparision of genes upregulated upon *eft-2* RNAi in N2 and *zip-2(tm4248)* animals. A separate Excel file is provided.

Table S9: Comparision of genes upregulated upon *eif-2α* RNAi and *eft-2* RNAi in N2 animals. A separate Excel file is provided.

Table S10: Quantitative reverse transcription-PCR primers used in the study

Gene name	Forward primer sequence (5'-3')	Reverse primer sequence (5'-3')
Pan-act	TCGGTATGGGACAGAAGGAC	CATCCCAGTTGGTGACGATA
irg-1	TCCGAAGACCGCACAAAAGT	TTTAGCCTCAAAGCCGGAGG
irg-2	ACTCAATTCGTGGCCAACCT	TGCCCAGTAACTTGTGTGGG

ugt-31	TCCCCAGACGGCTCTTCTAA	ACATCGGAGCATTACGTCCC
T16G1.5	GAATGACGCGCTGTTGAGTC	TTGGATGCAACTCGTGTGGT
Y51B9A.8	AATCACCTGTAACGGAGGGC	TGCAGCAATCACGAGCAGTA
cyp-14A5	ATCTTCTGCAGTTCCCAGCC	CTGCAGGTAGTGGTGTCCAG
oac-32	TGGAACCTGTCTTGGACTTTCC	TGTCCACGGAGACAAAAGCA
pgp-5	CCCTTGTTGCCATTTTCAAGC	CAAACGGTTGACACATACCAGT
E02C12.8	TGTCAAGAATCGCCTGCCTT	GCTGAGAGCTTATCTTCAACGC
clec-186	AAGCGAGTTGCAGACATCCA	CGACGGTAGCAAGGCTGTAT
clec-209	GCTACCGGAGCCTATTCCAC	GGCTCCCGAAGCTGGATAAA
dod-24	ACCGAGCCAGGAGGTTATCT	TTCTGTTGTCCGTCCCGATG
ilys-3	AAGAGGTGCGCGAATGATCT	CCTGTCCAGTTCCAGCACAT
ilys-5	TTCTGCTCCTTTCTGTCGCT	AGTATCCGCAGGAGAGGGAT
lys-1	GTTACCTCCCCAGCCAACTG	AGTCCGTATTGCTTGGCTCG
lys-7	CAGATCACGACCGGATGGAG	TCCACCGCTGTACACATTCC
lys-8	AAGTCATTGCTGAGGCTGCT	ACGGACAAAGACTGCCGAAT
